# Hyaluronic Acid Immersion Enhances Gamma-Ray-Irradiation Cross-Linking of the Fish-Derived Type I Collagen Membrane

**DOI:** 10.1021/acsomega.5c07582

**Published:** 2025-09-10

**Authors:** Vincent Irawan, Ryoma Furusho, Yoshihiro Kodama, Yuta Aida, Hayato Laurence Mizuno, Yasutaka Anraku, Toshiyuki Ikoma

**Affiliations:** † Chair of Bioseparation Engineering, TUM School of Engineering and Design, 9184 Technical University of Munich, Boltzmannstr. 15, 85748 Garching, Germany; ‡ Department of Materials Science and Engineering, School of Materials and Chemical Technology, 13290 Institute of Science Tokyo, 2-12-1 Ookayama, Meguro-ku, Tokyo 152-8550, Japan; § Taki Chemical Co., Ltd., 346 Miyanishi, Harima-cho, Kako-gun, Hyogo 675-0145, Japan

## Abstract

Fish-derived type I collagen is widely used in biomedical implants; however, its limited thermal and enzymatic stability necessitates cross-linking for clinical applications. This study investigated gamma-ray-induced cross-linking of collagen as a shape of membranes, with enhancement via polysaccharide-assisted immersion. Hydrated collagen membranes were irradiated at doses ranging from 15 kDa to 35 kGy to assess their dose-dependent effects. Irradiation at 15 kGy enhanced thermal and enzymatic stability, which declined at higher doses. To further enhance stability, the membranes were immersed in phosphate-buffered saline containing chondroitin sulfate (CS) or hyaluronic acid (HA) during irradiation at 25 kGy. HA-treated membranes showed a synergistic enhancement in thermal stability (*T*_onset_ > 50 °C, *T*_peak_ ∼ 54 °C) and enzymatic resistance, surpassing CS-treated and nonimmersed controls. MALDI-TOF mass spectrometry revealed the selective loss of irradiation-sensitive amino acid residues, indicating involvement in cross-linking and scission mechanisms. Tensile testing confirmed that the HA-treated membranes retained a tensile strength (∼1.7 MPa) under hydrated conditions, comparable to the irradiated-only controls. These findings demonstrate that HA-assisted gamma-irradiation at 25 kGy effectively enhances collagen cross-linking, improving its thermal and enzymatic stability while preserving its mechanical performance for biomedical applications.

## Graphical abstract

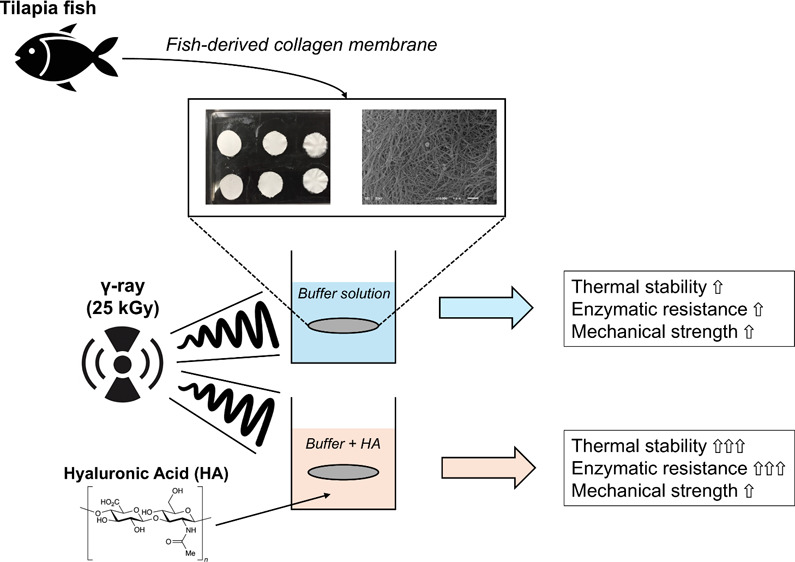

Fish-derived type I collagen is a fibrillar protein extensively utilized in medical applications owing to its biocompatibility, biodegradability, and regenerative capacity.^1–3^ It is often employed as a scaffolding material that can be implanted to support tissue regeneration^4^ while temporarily assuming a structural role at the implantation site.^5^ Collagen scaffolds are available in various forms, such as membranes for dental and dermal repair^6^ and sponges for musculoskeletal tissue regeneration.^7^ However, owing to its biological origin, collagen degrades rapidly under physiological conditions. To mitigate this issue, cross-linking is commonly applied before implantation to improve mechanical properties, thermal stability, and enzymatic resistance.^8^ Cross-linking strategies typically involve chemical methods, such as carbodiimide (CDI)-mediated coupling^8^ and physical techniques, including ultraviolet (UV)^9^ and gamma-ray irradiation.^10^ From a practical perspective, gamma-ray irradiation is particularly attractive because it can simultaneously induce cross-linking and sterilize collagen scaffolds, effectively merging two essential processing steps into one.^11^ This dual functionality offers a streamlined approach that is ideal for industrial and clinical applications. A standard dose of 25 kGy is generally accepted for sterilization without significantly impairing collagen-based devices.^12^ Despite its practicality, gamma-irradiation typically results in weaker cross-linking efficiency than chemical methods and may induce chain scission, potentially compromising the structural integrity of collagen.^12^

Our group has developed an optimized protocol to enhance gamma-ray-induced cross-linking of collagen membranes.^10^ This protocol involves immersing the membranes in phosphate-buffered saline (PBS) prior to and during irradiation. Under hydrated conditions, gamma-irradiation generates free radicals from water molecules, which subsequently react with amino acid residues to promote indirect cross-linking.^13^ Irradiation in the hydrated state is essential, as dry-state irradiation tends to favor chain scission,^14^ leading to significant degradation of collagen. Therefore, improving the efficiency of irradiation-induced cross-linking by optimizing PBS immersion is of particular interest.

Natural polysaccharides, such as chondroitin sulfate (CS) and hyaluronic acid (HA), are characterized by their unique functional groups and have been incorporated into collagen scaffolds to improve their bioactivity,^15^ mechanical stability,^16^ and cross-linking efficiency.^17^ In the context of gamma-irradiation, these polysaccharides can potentially improve the final properties of scaffolds in various ways. For instance, CS possesses antioxidant properties that may protect against chains scission,^18^ whereas HA can provide additional functional groups that contribute to cross-link formation.^17^ Based on these insights, we hypothesized that enriching the PBS immersion with CS or HA for gamma-irradiation could enhance the cross-linking efficiency and structural stability of collagen scaffolds. This study demonstrates the first attempt to improve irradiation-based cross-linking of collagen by modifying the solution parameter hydration used to immerse the collagen.

Building on this foundation, the current study aimed to examine the synergistic effects of CS or HA incorporation on the gamma-ray-induced cross-linking efficiency of collagen membranes. Initially, we evaluated the impacts of varying gamma-ray doses on the denaturation temperature and enzymatic degradation of collagen membranes, as indicators of cross-linking efficiency and structural stability. Subsequently, CS or HA was dissolved in PBS, and the collagen membranes were irradiated at a standard dose of 25 kGy. The experimental protocol is described in detail in the Supporting Information.

Figure 1A shows collagen hydrogels, characterized by a homogeneous and hydrated appearance. Upon dehydration, these hydrogels were converted into rigid and intact membranes, as depicted in Figure 1B. Scanning electron microscopy (SEM, Figure 1C) revealed that both noncross-linked (NXL) and gamma-irradiated (sample names abbreviated by dose) membranes, whether immersed in CS or HA during irradiation (CS + 25 kGy, HA + 25 kGy), maintained their characteristic fibrillar collagen architecture. The fibril diameters were 74 ± 17 nm (15 kGy), 72 ± 15 nm (20 kGy), 71 ± 15 nm (25 kGy), 70 ± 15 nm (30 kGy), 70 ± 18 nm (35 kGy), 77 ± 25 nm (CS + 25 kGy), and 75 ± 14 nm (HA + 25 kGy), closely matching 70 ± 15 nm of NXL as a control. This suggests that gamma-irradiation and polysaccharide immersion do not significantly alter fibrillar morphology, which is consistent with previous studies.^19^

**Figure 1:**
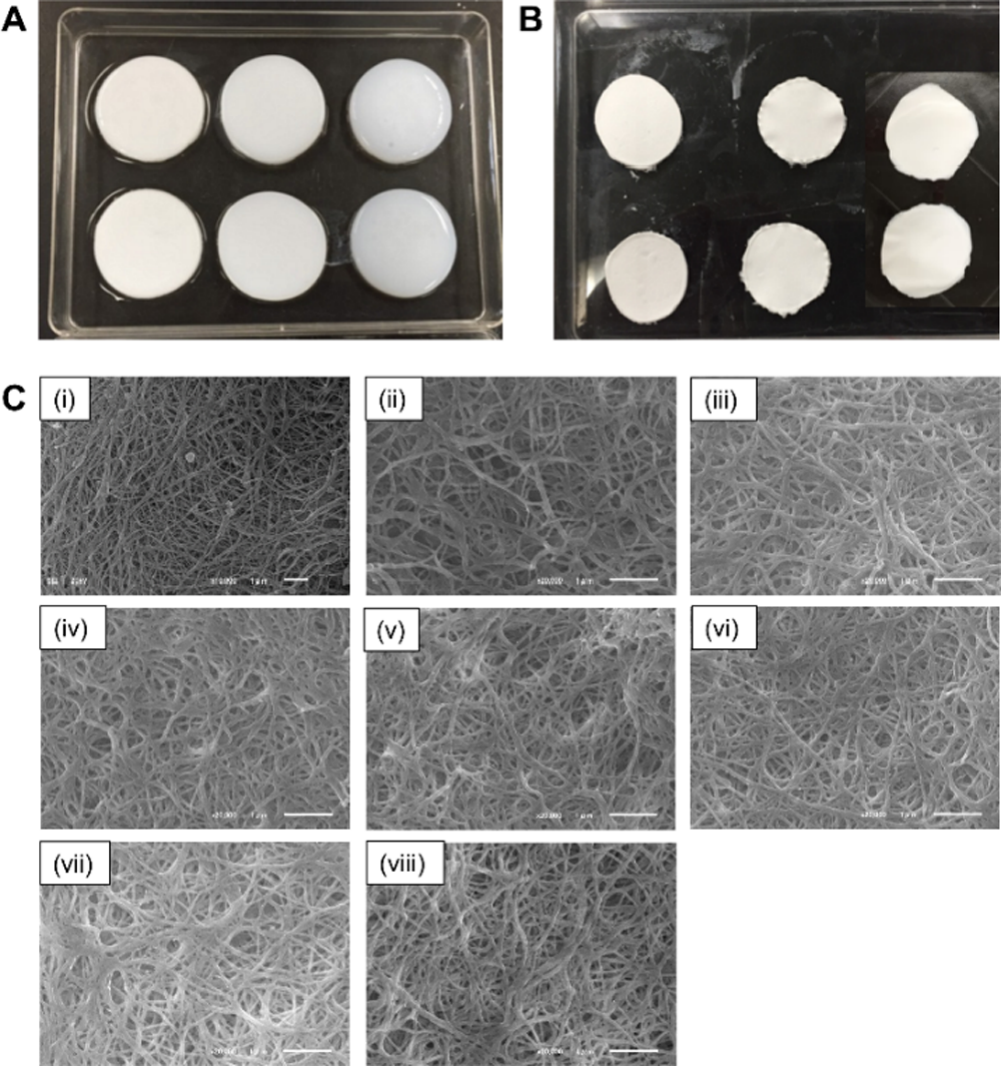
Macroscopic and microscopic characterization of collagen membranes before and after gamma-irradiation with or without polysaccharide treatment. (A) Hydrated collagen hydrogels prior to drying, (B) dried collagen membranes that maintain uniform shape and thickness, and (C) scanning electron microscopy images of collagen membranes under various conditions: (i) NXL, (ii) 15 kGy, (iii) 20 kGy, (iv) 25 kGy, (v) 30 kGy, (vi) 35 kGy, (vii) CS + 25 kGy, and (viii) HA + 25 kGy. Scale bar is 1 mm.

Attenuated total reflection Fourier-transform infrared (ATR-FTIR) spectroscopy (Figure 2) revealed molecular-level changes in collagen. The NXL spectrum (Figure 2i) showed characteristic amide I, II, and III bands at 1654, 1541, and 1229 cm^–1^, indicating a preserved triple-helix structure,^20^ along with a broad N–H stretching band at 3296 cm^–1^. Peaks at 2917 and 2848 cm^–1^ (CH_2_ and CH_3_ stretching)^21^ arise from side chains such as proline, lysine, and methionine. A doublet at 1080 and 1025 cm^–1^ reflects phenylalanine,^6^ while peaks at 1451, 1397, 1335, and 1281 cm^–1^ represent CH_3_/CH_2_ deformations and C–N stretching. A prominent 838 cm^–1^ peak corresponds to C–C stretching of proline/hydroxyproline rings.^6^ Following irradiation, the peaks at 2917, 2848, and 838 cm^–1^ and the phenylalanine peaks at 1025/1080 cm^–1^ gradually decreased, indicating amino acid structural changes likely due to chain scission or cross-linking. FTIR spectra of the polysaccharide-treated membranes (Figure 2vii,viii) exhibited no discernible differences, which can be attributed to overlapping absorption bands with collagen^22,23^ and the limited binding amount. The deconvolution of the amide I bands (Figure S2) highlighted two peaks: 1657 and 1630 cm^–1^, and the 1657/1630 peak area ratio is a hallmark of collagen denaturation.^24,25^ The full width at half-maximum (fwhm) for each sample is as follows: 0.65 ± 0.01 (NXL), 0.64 ± 0.01 (15 kGy), 0.67 ± 0.02 (20 kGy), 0.60 ± 0.04 (25 kGy), 0.59 ± 0.09 (30 kGy), 0.64 ± 0.04 (35 kGy), 0.82 ± 0.04 (25 kGy + CS), and 0.76 ± 0.05 (25 kGy + HA). This ratio remained at approximately 0.65 for NXL and samples irradiated up to 20 kGy but dropped to ∼ 0.60 for doses above 25 kGy. Although this indicates increased denaturation in some collagen regions with higher irradiation doses, the differences were not statistically significant, suggesting that the triple-helical structure of collagen remained largely intact, consistent with the findings of Inoue et al.^26^ The fwhm increased in 25 kGy + CS (0.82 ± 0.04) and 25 kGy + HA (0.76 ± 0.05), attributed to C = O band overlapping with amide I. Therefore, the impact of polysaccharide immersion on irradiation-induced denaturation remains unclear, though some CS and HA appear to bind to collagen.

**Figure 2:**
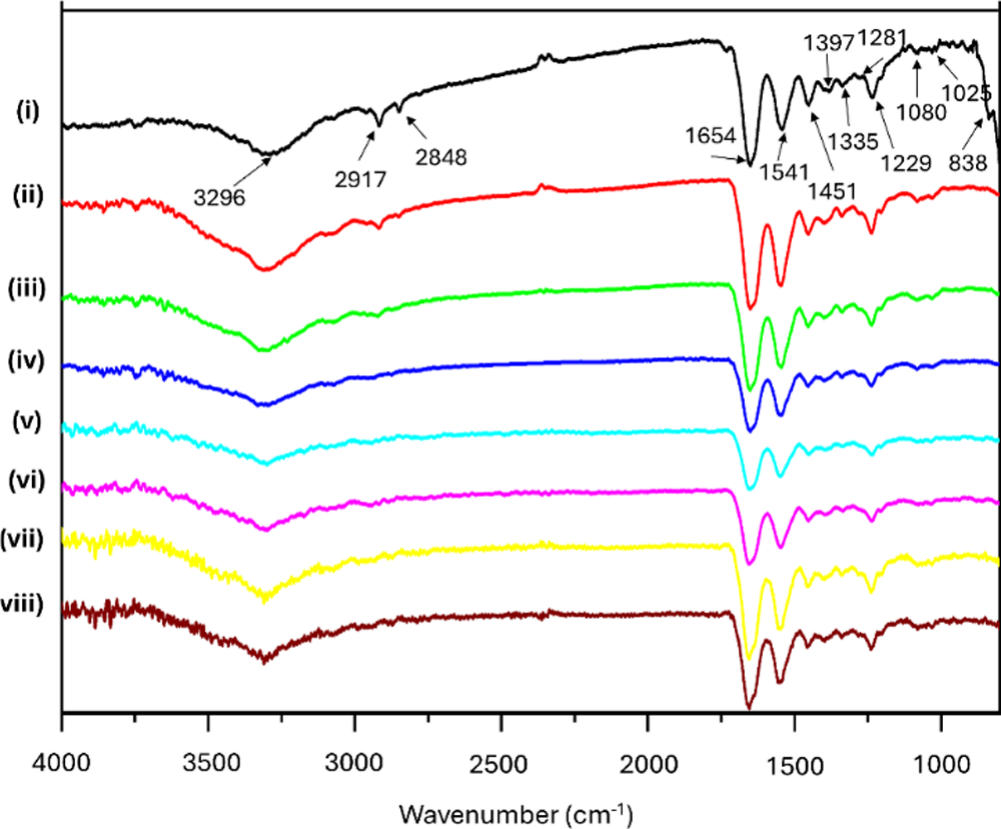
Attenuated total reflectance Fourier-transform infrared spectra of collagen membranes subjected to different irradiation doses (i–vi) and immersion conditions (vii, viii): (i) NXL, (ii) 15 kGy, (iii) 20 kGy, (iv) 25 kGy, (v) 30 kGy, (vi) 35 kGy, (vii) CS + 25 kGy, and (viii) HA + 25 kGy.

To confirm irradiation-induced changes in amino acids, matrix-assisted laser desorption/ionization time-of-flight mass spectrometry (MALDI-TOF) spectrometry (Figure 3) was performed on collagen membranes before and after irradiation.^27^ The NXL membrane exhibited distinct peaks corresponding to five Gly–Pro–X tripeptides, where X is isoleucine (GPI), threonine (GPT), arginine (GPR), lysine (GPK), or histidine (GPH).

**Figure 3:**
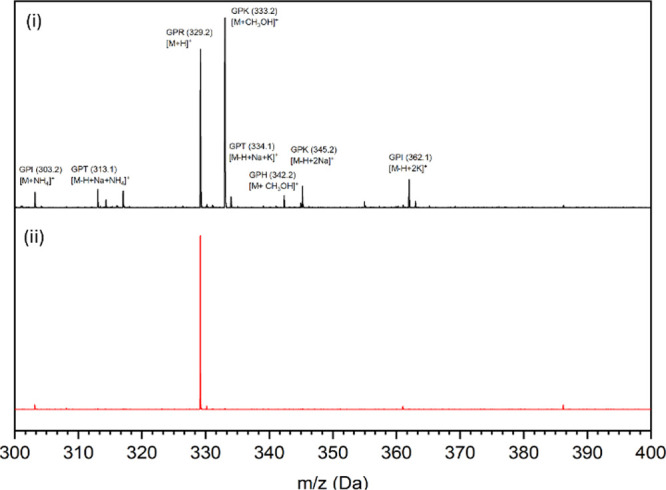
MALDI-TOF profile of collagen membranes before and after gamma-irradiation. Distinct peaks corresponding to specific tripeptide fragments (Gly–Pro–X, where X is isoleucine (GPI), threonine (GPT), arginine (GPR), lysine (GPK), or histidine (GPH)) are noted in the figure. Each tripeptide is detected as a positively charged ion, forming various adducts (e.g., H^+^, Na^+^, K^+^, CH_3_OH, and NH_4_^+^), which are indicated in square brackets. The corresponding mass-to-charge ratios (*m*/*z*) of the ionized species are given in parentheses.

These tripeptides are degradation products resulting from enzymatic cleavage by collagenase A.^27^ Notably, these amino acid residues have been previously identified as irradiation-sensitive.^28^ Following gamma-irradiation, the corresponding peaks for isoleucine, threonine, lysine, and histidine were significantly reduced or absent, indicating their susceptibility to irradiation-induced cleavage or potential involvement in cross-linking reactions. Meanwhile, the arginine peak was retained. Gamma-irradiation generates reactive species (•OH, •H, e_a_q^–^, and O_2_^–^) through water radiolysis,^13^ causing peptide scission or covalent cross-linking via radical recombination. Amino acids like histidine or phenylalanine can stabilize unpaired electrons,^13,29^ promoting cross-linking, while nonconjugated residues such as isoleucine and threonine are more prone to scission.^28^ Lysine, though not radical-stabilizing, may facilitate cross-linking through its ε-amino group.^30^ These observations highlight the complex interplay between scission and cross-linking pathways in gamma-irradiated collagen, involving a broad range of amino acids, as supported by both FTIR and MALDI-TOF analyses.

Differential scanning calorimetry curves (DSC, Figure 4A) for NXL collagen showed a gradual decline in heat flow beginning at 38.3 ± 1.6 °C (*T*_onset_), followed by a broad, asymmetric endothermic peak at 51.7 ± 2.2 °C (*T*_peak_). These features reflect the heterogeneous thermal behavior caused by variability under hydration, fibril compaction, and collagen molecule interactions.^31,32^ *T*_onset_ marks the initial disruption of the triple-helix conformation, whereas *T*_peak_ reflects the temperature at which the majority of helices are fully denatured into random coils.^32^ *T*_onset_ and *T*_peak_ for each sample are summarized in Figure 4B. At 15 kGy, the curves became sharper and more symmetrical, and their *T*_onset_ increased to 47.0 ± 0.9 °C, suggesting that cross-linking induced by irradiation enhanced the thermal stability of the triple helices, likely by reducing molecular mobility. Conversely, *T*_peak_ decreased to 48.5 ± 0.9 °C, implying that although denaturation begins at a higher temperature, the complete helix-to-coil transition occurs over a narrower temperature range and at a lower peak temperature. This shift may result from irradiation-induced partial denaturation and chain scission, which reduce the thermal energy of denaturation. This finding contrasts sharply with the results reported by Hara et al., who observed a diminished endothermic peak and decreases of *T*_onset_ and *T*_peak_ upon irradiation of fibrillar collagen gels.^19^ This discrepancy likely stems from differences in hydration and molecular mobility between the hydrogel and membrane states. In hydrogels, high water content and collagen mobility promote reactive species formation and chain scission, reducing cross-linking efficiency. In contrast, membrane formation involves dehydration and fibril densification, limiting radical diffusion and scission while promoting dense interfibrillar cross-linking, enhancing thermal and enzymatic stability. As the irradiation dose increased beyond 15 kGy, both *T*_onset_ and *T*_peak_ declined progressively, implying that chain scission began to surpass the cross-linking effects at higher irradiation doses. This is an effect commonly seen in physical cross-linking methods like UV or dehydrothermal treatment.^9,33^ Immersion in polysaccharides during irradiation dramatically altered these trends. Samples immersed in HA and irradiated at 25 kGy exhibited *T*_onset_ values above 50 °C and a *T*_peak_ of 54 °C, representing substantial thermal stabilization

**Figure 4:**
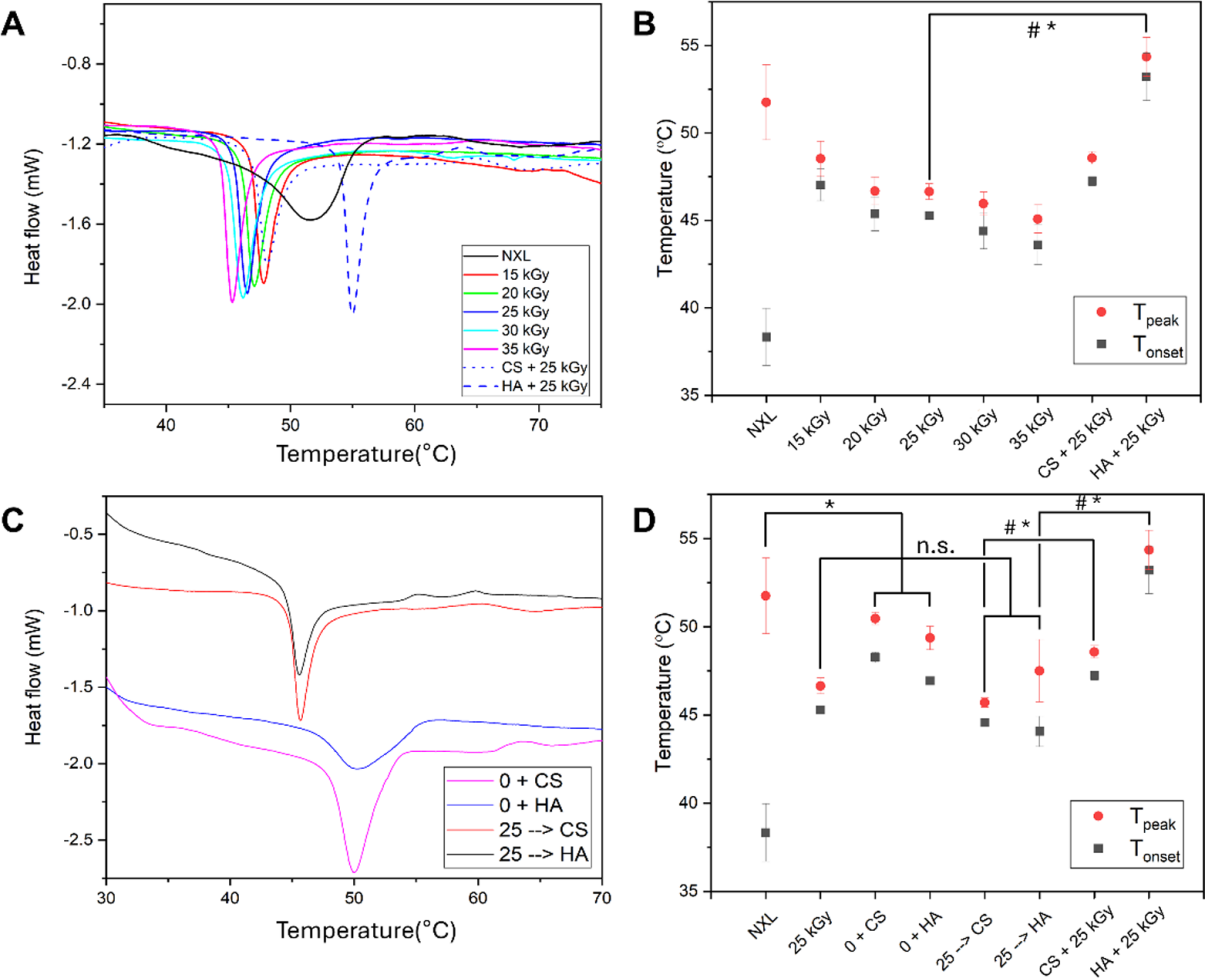
Thermal properties and the temperatures of onset and denaturation of the collagen membrane. (A, C) DSC curve for the collagen membranes irradiated in (A) different doses and in the presence of HA and CS, and in (C) immersion with irradiation and sequential immersion and irradiation. (B, D) Summary of onset and denaturation temperatures for DSC curves in (A, C). *n* = 3, mean ± standard deviation. Statistical comparisons were performed using a two-tailed Student’s *t* test, with *p* < 0.05 considered statistically significant. # and * indicate a statistically significant difference in *T*_peak_ and *T*_onset_, respectively, compared to the 25 kGy group (*p* < 0.05). n.s. is nonsignificant.

Next, we determined whether the thermal resistance enhancement stems from polysaccharide immersion or a synergistic effect with gamma-irradiation (Figure 4C,D). In Figure 4C, collagen membranes were treated with either (i) immersion in CS or HA without irradiation (0 + CS or HA), or (ii) gamma-irradiation at 25 kGy followed by immersion (25 kGy → CS or HA). Immersion in CS or HA alone significantly increased *T*_onset_ compared to that in NXL, but no further enhancement was seen with subsequent irradiation, suggesting that CS and HA may form stabilizing complexes with collagen. However, irradiation appears to disrupt these interactions or introduce competing degradation. Taken together, only HA-immersed membranes showed a synergistic effect with gamma-irradiation, despite the similar functional group chemistries of HA and CS. HA, owing to its higher molecular weight and extended conformation, may induce a macromolecular crowding effect,^34^ promoting closer packing of collagen fibrils and more effective cross-linking. In contrast, the expected protective role of CS against irradiation-induced degradation was apparently absent under these conditions. The collagen membranes were subjected to enzymatic degradation using collagenase A (Figure 5A).

**Figure 5:**
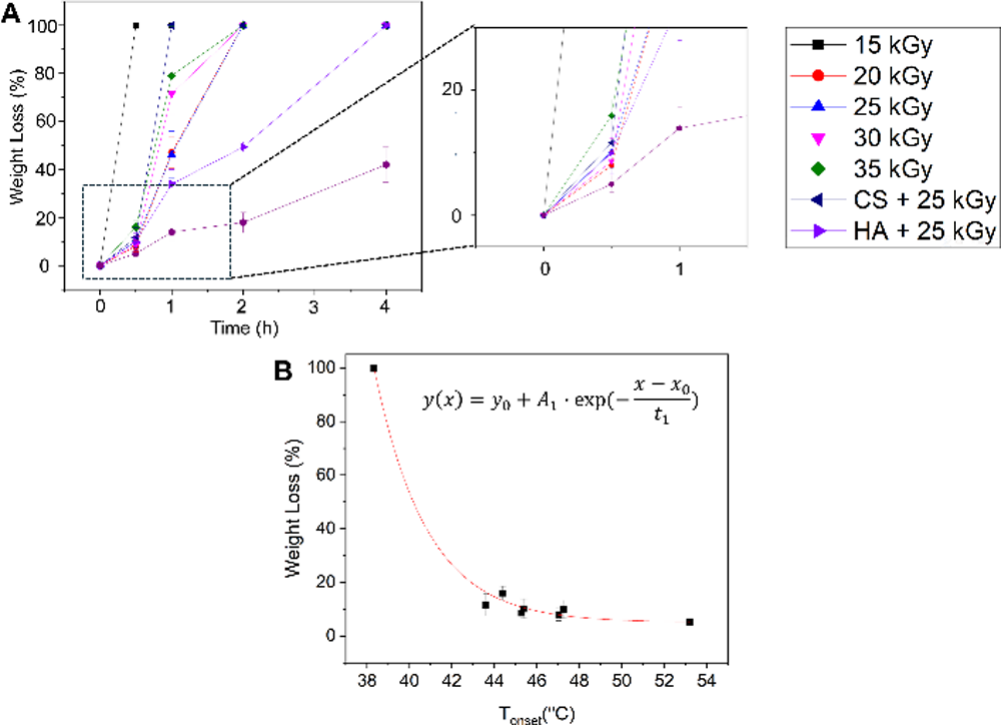
Enzymatic stability of collagen membranes subjected to gamma-irradiation and polysaccharide treatment. (A) Time-dependent enzymatic degradation of collagen membranes incubated in collagenase A solution. (B) Weight loss at 0.5 h plotted against *T*_onset_ fitted with the exponential decay model (where *A*_1_ = 100%, *t*_1_ = 2.48 ± 0.23 °C, *x*_0_ = 38.21 °C ± 0.03 °C, and *y*_0_ = 5.01 ± 1.07% (*R* ^2^ = 0.999), and linear model (*a* = 51.33 ± 10.55 and *b* = −0.86 ± 0.21, *R* ^2^ = 0.77). Data are presented as the mean ± standard deviation (*n* = 3).

NXL membranes completely degraded within 30 min. In contrast, irradiated membranes (e.g., 15 kGy) exhibited significantly improved resistance, losing only ∼20% of their initial weight after 0.5 h. Notably, the CS/HA + 25 kGy membranes exhibited even higher resistance, with only ∼10 and ∼3% degradation, respectively. At 1 h, degradation varied by doses: 15 and 20 kGy membranes lost ∼45% mass, while 25 and 30 kGy membranes degraded by 70–80%. All 35 kGy membranes were fully degraded within 1 h. However, the CS + 25 kGy degraded by ∼45%, and HA + 25 kGy by ∼12% at 1 h. After 2 h, the weight loss for HA-treated membranes slightly increased to ∼15%, whereas that for CS-treated membranes by ∼50%. At 4 h, only the HA-treated membranes remained intact. The reduced weight loss in HA-treated samples results from enhanced cross-linking efficiency and a steric barrier formed by the polysaccharide, limiting collagenase access to cleavage sites.^35^ The enhanced enzymatic stability in HA-treated membranes cannot be attributed solely to nondegradable HA, as all CS- or HA-treated membranes had similar wet weights (∼1.8 mg), indicating minimal polysaccharide binding relative to collagen mass.

To understand the relation of thermal stability and enzymatic resistance, the weight loss after 0.5 h was plotted against *T*_onset_ (Figure 5B), with the fitting details provided in the figure and caption. *T*_onset_, reflecting the initial destabilization of the collagen triple helix, was used as an indirect measure of cross-linking, in contrast to *T*_peak_, which indicates the final denaturation temperature and is less sensitive to early structural changes. A strong exponential decay relationship was observed between weight loss and *T*_onset_ (Figure 5B), as described by the fitted equation. Here, *y*(*x*) represents the weight loss (%) as a function of *T*_onset_. The parameter *y*_0_ denotes the residual weight loss, while *A*_1_ is the amplitude of decay, reflecting the maximum weight loss in thermally unstable samples. *x*_0_ at 38.2 °C indicates the onset of denaturation temperature taken from noncross-linked collagen. Finally, *t*_1_ is the decay constant, representing how sensitive the weight loss decreases with increasing *T*_onset_. This correlation can be interpreted that gamma-irradiation stabilizes the collagen triple-helix via cross-linking, leading to increased *T*_onset_ and greater resistance to enzymatic degradation. However, the diminishing loss at higher *T*_onset_ may indicate that nonenzymatic mechanisms also contribute to weight loss.

Mechanical testing was performed under hydrated conditions, and it was revealed that the NXL membranes lacked sufficient integrity for tensile testing. The irradiated samples exhibited a linear elastic response followed by abrupt fracture without plastic deformation (Figure 6A). At 15 kGy, the Young’s modulus was 3.4 ± 0.4 MPa. When the irradiation dose was increased to 20, 25, and 30 kGy, the modulus remained relatively stable at 3.4 ± 0.3, 3.7 ± 0.4, and 3.5 ± 0.6 MPa, respectively. However, a slight increase was observed at 35 kGy, where the modulus reached 4.3 ± 0.3 MPa. In contrast, polysaccharide treatment led to modulus reductions of 1.9 ± 0.1 MPa (CS + 25 kGy) and 3.1 ± 0.2 MPa (HA + 25 kGy). The ultimate tensile strength (UTS) was unchanged between 1.5–2.0 MPa in the irradiated-only groups, while the CS + 25 kGy membranes dropped significantly to 0.9 ± 0.1 MPa (*p < 0.05), and HA + 25 kGy was 1.7 ± 0.1 MPa. In collagen fibril-based materials, the mechanical properties depend largely on the interfibrillar cross-links and alignment.^36^ The minimal increase in stiffness with the irradiation dose suggests that irradiation-generated free radicals have limited access to interfibrillar regions and primarily act within the intrafibrillar domains. This may result from (1) the short half-life and limited diffusion of free radicals and (2) the lower mobility of collagen fibrils in the membrane. These factors are also likely to contribute to the high variability in the mechanical properties of the irradiated membranes. While 35 kGy irradiation resulted in an increase in stiffness, this did not translate into higher UTS, suggesting that the membrane became more rigid but not necessarily more resistant to mechanical failure. Treatment with CS or HA reduced stiffness and tensile strength, likely due to the increase in hydration from the adsorption of CS or HA.

**Figure 6:**
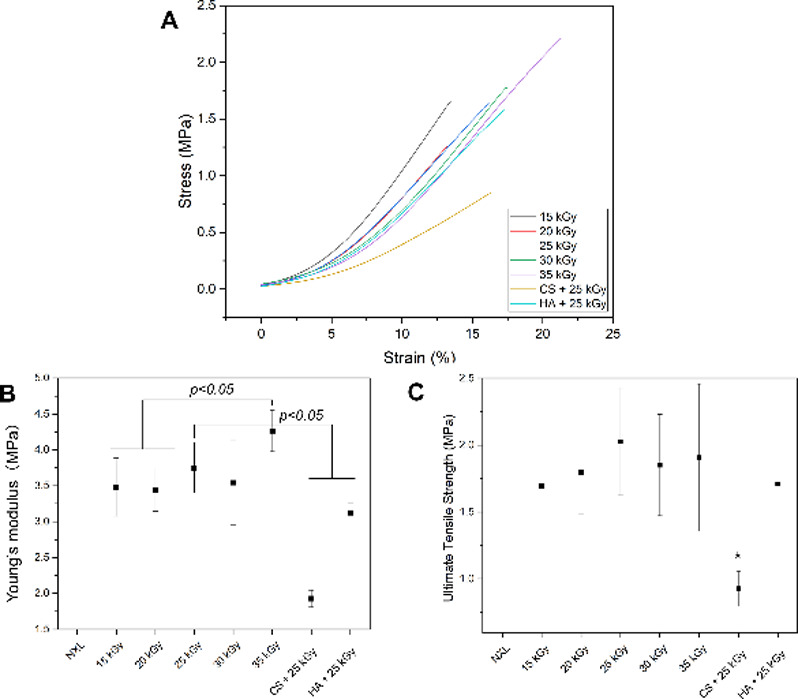
Mechanical properties of collagen membranes subjected to gamma-irradiation and polysaccharide treatment. (A) Representative stress–strain curve of the collagen membrane. (B) Young’s modulus and (C) ultimate tensile strength (UTS) of collagen membranes in hydrated conditions. Data were expressed as mean ± standard deviation, *n* = 5. Statistical comparisons were performed.

This study demonstrates that modifying the gamma-irradiation environment with polysaccharides can substantially affect the cross-linking efficiency and functional performance of collagen membranes. Preirradiation immersion in HA significantly enhanced thermal stability and enzymatic resistance, which can be attributed to its synergistic interaction with gamma-irradiation cross-linking. This enhancement is likely due to HA-mediated macromolecular interactions and their impact on cross-linking dynamics. Although CS also contributed to stabilization, its effects were less pronounced and did not exhibit a synergistic effect with irradiation. These findings promote the integration of HA-assisted irradiation as a scalable and potentially dual-purpose strategy for cross-linking and sterilizing collagen scaffolds, providing a foundation for extending this method to other biomaterial systems.

A limitation of this study is that we did not quantify the amount of CS and HA uptake. Unlike collagen, CS and HA do not contain active cell-binding sites. Therefore, excessive uptake of CS and HA may potentially reduce cell activity in a concentration-dependent manner. Future studies should include a systematic evaluation of the cytocompatibility of the current scaffolds.

## Supplementary Material



## Notes

Materials and methods; representative FTIR deconvolution image; and summary of deconvoluted peaks (PDF)

## Abbreviations

HA: hyaluronic acid
CS: chondroitin sulfate
PBS: phosphate-buffered saline
ATR-FTIR: attenuated total reflectance Fourier-transform infrared spectroscopy
MALDI-TOF: matrix-assisted laser desorption/ionization time-of-flight
DSC: differential scanning calorimetry
*T*_onset_: onset temperature
*T*_peak_: peak denaturation temperature
UTS: ultimate tensile strength
MPa: megapascal
SEM: scanning electron microscopy
NXL: noncross-linked
CDI: carbodiimide
UV: ultraviolet
fwhm: full width at half-maximum
