# Supplementary material for: Hyaluronic Acid Immersion Enhances Gamma-Ray-Irradiation Cross-Linking of the Fish-Derived Type I Collagen Membrane
Source: ACS Omega. 2025 Sep 10;10(37):43273–9. doi: 10.1021/acsomega.5c07582 (PMC12461391; doi:10.1021/acsomega.5c07582)
Supplement: Supplementary file 1 [file ao5c07582_si_001.pdf]

Hyaluronic Acid Immersion Enhances Gamma-Ray-Irradiation Crosslinking of Fish-derived Type I Collagen Membrane

Vincent Irawan[1], Ryoma Furusho[2], Yoshihiro Kodama[3], Yuta Aida[2], Hayato Laurence Mizuno[2], Yasutaka Anraku[2], and Toshiyuki Ikoma[2]*

Section S1. Materials and Methods

Materials

Tilapia fish scale type I collagen (Cellcampus FD-08G) was provided by Taki Chemical Co. Ltd. Chondroitin sulfate sodium salt (ND-K, 20,000 Da M.W., Seikagaku Co. Ltd) and hyaluronic acid sodium salt from Rooster Comb (for biochemistry, Fujifilm Wako Pure Chem. Co.) were used without further purification. 2,5-Dihydroxybenzoic acid (DHB, 99.0%), and 0.1% trifluoroacetic acid (TFA, 99.0%) were purchased from TCI Chemicals, Japan. Phosphate buffered saline powder (PBS), Dulbecco's phosphate-buffered saline tablet without calcium and magnesium (DBPS), TES (N-[Tris(hydroxymethyl)methyl]-2-aminoethanesulfonic acid, > 99%), $CaCl_2$ (ACS Reagent > 96%), collagenase A (EC 3.4.24.3 from *Clostridium histolyticum*), Tris(hydroxymethyl)aminomethane hydrochloride (ACS Reagent, Purity < 98%, Tris-HCl) and ethylenediaminetetraacetic acid disodium salt dihydrate (ACS Reagent 99-101%, EDTA) were purchased from Sigma Aldrich.

Methods

Fabrication of Collagen Membranes and the Exposure to Gamma-Ray Irradiation

Collagen membranes were prepared using the following procedure: a 1.1 wt% collagen solution in hydrochloric acid (pH 3.0) was mixed with 10 × DPBS at a 9:1 volume ratio at 4°C, then cast into cylindrical molds with a diameter of 20 mm and a height of 2.5 mm, and incubated overnight at 25°C to induce collagen self-assembly. The resulting hydrogels were dehydrated using a graded ethanol series, sandwiched between polystyrene films, and air-dried to remove residual ethanol[1]. The dried membranes were soaked in DPBS and irradiated with gamma rays from a Cobalt-60 source at doses ranging from 15 to 35 kGy. Samples were labeled as NXL (non-crosslinked) or according to the irradiation dose (e.g., 15 kGy, 20 kGy, 25 kGy, 30 kGy, 35 kGy). Additional membranes were immersed in DPBS containing either 0.5 wt% chondroitin sulfate (CS) or 0.5 wt% hyaluronic acid (HA), irradiated at 25 kGy, and designated as 25 kGy + CS and 25 kGy + HA, respectively.

Microstructure and Chemical Analysis of Collagen Membranes

Collagen membranes after gamma-ray irradiation were dehydrated using a graded ethanol series, followed by solvent exchange with tert-butyl alcohol[1]. The samples were then freeze-dried overnight. To examine the

fibrous structures, the dehydrated samples were transversely cut and mounted on carbon tape. Platinum sputter-coating was applied prior to scanning electron microscopy (SEM; JSM-6510, JEOL Co., Japan). Imaging was conducted at an accelerating voltage of 20 kV and a working distance of 10 mm. A total of 100 fibrils were measured for sample type (n = 3). The results are presented as mean ± standard deviation. The chemical characterization of the collagen membranes was performed using Attenuated Total Reflectance Fourier-Transform Infrared (ATR-FTIR) spectroscopy (DR-81, JASCO) with a mercury cadmium telluride (MCT) detector cooled with liquid nitrogen. Spectra were collected in the range of 4000–800 cm^{-1} at a resolution of 2.0 cm^{-1} and an accumulation of 156, and the background was calibrated with a gold mirror standard.

Denaturation Degree via FTIR Peak Deconvolution

To quantify the degree of denaturation, FTIR peaks at 1675 cm^{-1} (amide I) were deconvoluted using the FitPeaks function in OriginLab software. The deconvolution process included manual baseline correction, peak selection, application of Gaussian fit, and curve fitting. A coefficient of determination (Adjusted R^2) greater than 0.98 was used as the threshold for a good fit, as summarized in Table 1. Two major deconvoluted peaks were identified at 1654 and 1630 cm^{-1}, and the full width at half maximum (FWHM) ratio of these peaks was used as an indicator of denaturation[2].

Table 1. Adjusted R^2 for deconvoluted 1675 cm^{-1} peak

Sample name	n = 1	n = 2	n = 3
NXL	0.992	0.998	0.998
15 kGy	0.998	0.998	0.998
20 kGy	0.999	0.998	0.999
25 kGy	0.998	0.996	0.998
30 kGy	0.998	0.989	0.999
35 kGy	0.998	0.996	0.999
25kGy + CS	0.997	0.998	0.998
25 kGy + HA	0.999	0.999	0.999

Identification of Irradiation-Affected Amino Acids via MALDI-TOF

Amino acid residues involved in gamma-ray-induced crosslinking were analyzed using matrix-assisted laser desorption/ionization time-of-flight mass spectrometry (MALDI-TOF; UltrafleXtreme, Bruker) equipped with a 355 nm Nd:YAG laser. This method followed a previously reported protocol[3]. Measurements were performed in the positive ion reflector mode using delayed extraction conditions. The extraction voltage was 20 kV, and gated matrix suppression was applied. Each spectrum was obtained by averaging 100 laser shots with random laser targeting to minimize sample heterogeneity.

A matrix for positive ion detection was prepared by mixing 0.5 M DHB solution in methanol and 0.1% TFA. Matrix and analyte were mixed in a 1:1 volume ratio. Non-irradiated and irradiated (25 kGy) collagen membranes (1 mg) were dissolved in 200 μL of 50 mM TES buffer (pH 7.4) containing 0.36 mM $CaCl_2$ to

achieve a concentration of 5 mg/mL. After confirming dissolution visually, the samples were heat-denatured at 95ºC for 10 min, followed by digestion with 0.1 mg/mL collagenase A (4 μL) at 37ºC for 24 h. The digests were mixed with the matrix at a 1:1 ratio. From the mixture of digest and matrix, 1 μL was spotted onto a MALDI target plate. The preferred cleavage site of collagenase A is between ~Pro-X and Gly-Pro-Y, where "X" is most often a neutral amino acid and "Y" can be any non-specific amino acid residue. Hence, the analysis of the *m*/z of the analyte that was degraded by collagenase was focused on identifying the possible tripeptide of Gly-Pro-Y in the *m*/z range of 300-400. The spectra were analyzed using open-source OpenMS software.

Thermal Analysis of Collagen Membranes

Differential scanning calorimetry (DSC; TAS-100, Rigaku Co., Tokyo, Japan) was used to evaluate the thermal behavior of the collagen membranes. Dehydrated membranes (2–3 mg) were rehydrated overnight in DPBS. Excess surface liquid was removed with KimWipes before sealing the samples in aluminum pans. DPBS was used as a reference. Measurements were conducted from 25 to 80ºC at a heating rate of 2 ºC /min in air. The onset temperature was determined by extrapolating the tangent at the beginning of the endothermic peak to intersect with the baseline. The peak temperature was defined as the temperature at the minimum endothermic peak. Data analysis was performed using OriginLab software.

Tensile Testing of Collagen Membranes

The mechanical properties of the collagen membranes were assessed via tensile testing using a Texture Analyzer (TA-XT2, Stable Micro Systems; $n = 5$). The samples were shaped into dumbbell-like specimens with a gauge length of 10 mm. The samples were dehydrated and re-equilibrated in PBS for 20 min. The samples were pulled at a rate of 2.0 mm/min. Young's modulus was calculated from the linear region of the stress−strain curve, and the ultimate tensile strength (UTS) was determined as the stress at break.

Enzymatic Degradation of Collagen Scaffolds

The enzymatic degradation behavior of collagen membranes was tested using collagenase A. Ethanol-dehydrated samples (8 mm diameter) were prepared ($n = 3$ per condition per time point). The sample weights were 1.5−1.7 mg. For the degradation test, each collagen membrane was placed in a 24-well plate containing 250 μL of 0.2 M Tris-HCl buffer (pH 7.4) and 250 μL of 0.1 M $CaCl_2$, and incubated at 37ºC for 1 h. Following pre-incubation, 500 μL of collagenase A solution (50 U/mL in 0.1 M Tris-HCl) was added to each well. After 1 h of enzymatic digestion at 37ºC, the reaction was terminated by adding 100 μL of 0.25 mM EDTA. The samples were subsequently washed twice with 2.5 mM EDTA and three times with distilled water to remove residual enzyme and buffer components. The degradation rate was calculated based on the mass loss using Equation (1):

$$\text{Mass loss (\%)} = \frac{[\text{Initial mass} - \text{final mass}]}{\text{Initial mass}} x\, 100 \qquad (1)$$

Reference

1. Irawan, V., Kajiwara, D., Nakagawa, Y. & Ikoma, T. Fabrication of mechanically robust bilayer membranes of hydroxyapatite/collagen composites. *Mater. Lett.* **291**, 129514 (2021).
2. Payne, K. J. & Veis, A. Fourier transform ir spectroscopy of collagen and gelatin solutions: Deconvolution of the amide I band for conformational studies. *Biopolymers* **27**, 1749–1760 (1988).
3. Nimptsch, A. *et al.* Quantitative analysis of denatured collagen by collagenase digestion and subsequent MALDI-TOF mass spectrometry. *Cell Tissue Res.* **343**, 605–617 (2011).

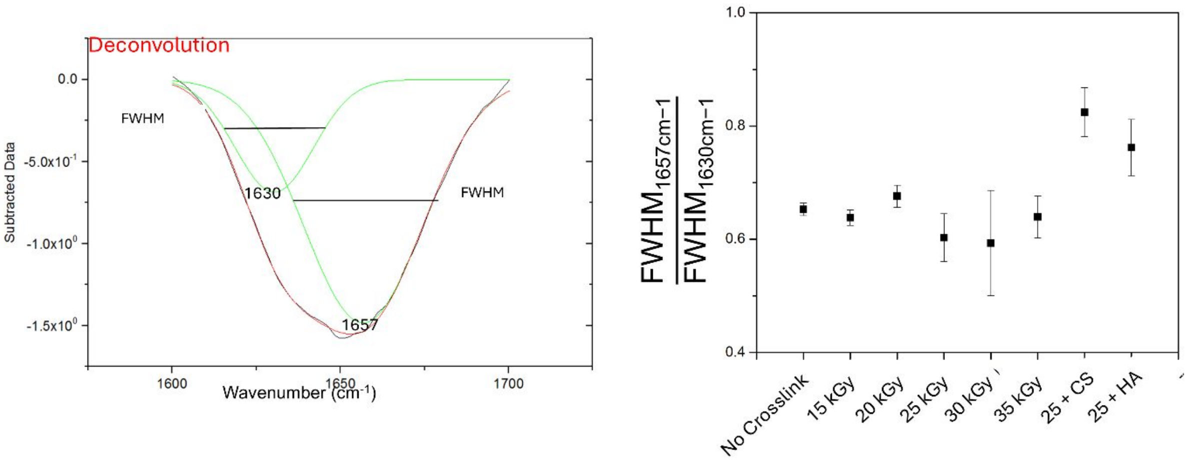

Fig. S2. Deconvolution method of the peak at 1654 cm-1 (left) and the ratio of $\frac{\text{FWHM}_{1657\text{cm-1}}}{\text{FWHM}_{1630\text{cm-1}}}$ for the collagen samples of increasing dose and polysaccharide immersed (right).
